# The Metabolic Effects of Oats Intake in Patients with Type 2 Diabetes: A Systematic Review and Meta-Analysis

**DOI:** 10.3390/nu7125536

**Published:** 2015-12-10

**Authors:** Qingtao Hou, Yun Li, Ling Li, Gaiping Cheng, Xin Sun, Sheyu Li, Haoming Tian

**Affiliations:** 1 Department of Endocrinology and Metabolism, West China Hospital, Sichuan University, Chengdu 610041, China; qingtao1990@sina.com; 2 Department of Endocrinology and Metabolism, The Third People’s Hospital of Chengdu, Chengdu 610031, China; lyhelen37@126.com; 3 Chinese Evidence-based Medicine Center, West China Hospital, Sichuan University, Chengdu 610041, China; ebmliling@hotmail.com (L.L.); sunx79@hotmail.com (X.S.); 4 Department of Clinical Nutrition, West China Hospital, Sichuan University, Chengdu 610041, China; hellochgp@163.com

**Keywords:** oats, β-glucan, type 2 diabetes mellitus, glycemic control, cholesterol, systematic review, meta-analysis

## Abstract

The present study aimed to comprehensively assess if oats intake is beneficial for diabetic patients. The literature search was conducted in PubMed database up to 23 August 2015. Fourteen controlled trials and two uncontrolled observational studies were included. Compared with the controls, oats intake significantly reduced the concentrations of glycosylated hemoglobin A1c (HbA1c) (MD, −0.42%; 95% CI, −0.61% to −0.23%), fasting blood glucose (FBG) (MD, −0.39 mmol/L; 95% CI, −0.58 to −0.19 mmol/L), total cholesterol (TC) (MD, −0.49 mmol/L; 95% CI, −0.86 to −0.12 mmol/L), low-density lipoprotein cholesterol (LDL-C) (MD, −0.29 mmol/L; 95% CI, −0.48 to −0.09 mmol/L). Oatmeal significantly reduced the acute postprandial glucose and insulin responses compared with the control meal. The present study has revealed a beneficial effect of oats intake on glucose control and lipid profiles in type 2 diabetic patients. Further investigations of oats intake in patients with type 1 diabetes and the safety of oats consumption are required.

## 1. Introduction

Type 2 diabetes is a common chronic disease with great global health and economic burden. The prevalence is still increasing due to lifestyle changes, especially in developing countries [1,2]. Diabetic education, nutrition therapy, physical activity, pharmacotherapy and glucose monitoring are key components of diabetes management. Lifestyle intervention including diet control is recommended as the fundamental approach for all patients with type 2 diabetes. Diabetic patients are suggested to consume at least the amount of fibers and whole grains recommended for the general public, which is 14 g fiber/1000 kcals daily or about 25 g/day for adult women and 38 g/day for adult men [3]. Dietary fibers promote one or more of the beneficial effects such as laxation, reduction in blood lipids, modulation of blood glucose due to their non-digestibility in the small intestine and fermentation in the colon. Oats are a good source of soluble dietary fiber rich in β-glucan, which is considered as a bioactive component in reducing postprandial glucose and insulin responses, improving insulin sensitivity, maintaining glycemic control and regulating blood lipids [4,5,6,7]. The United States Food and Drug Administration (FDA) suggested that the consumption of 3 g or more per day of β-glucan from oats or barley may reduce the risk of coronary heart disease [8].

A number of studies have reported the beneficial metabolic effects of oats or β-glucan on people with and without type 2 diabetes [9,10,11,12]. A modified diet with β-glucan from oats was reported to be superior to the American Diabetic Association’s diet in improving metabolic and anthropometric profiles in well controlled type 2 diabetic patients: larger decreases in glycosylated hemoglobin A1c (HbA1c), weight and body mass index (BMI); greater increase in high-density lipoprotein cholesterol (HDL-C) [9]. A high dose of barley β-glucan supplement (6.31 g β-glucan) improved the glucose and insulin responses when added to a high-carbohydrate food in lean, healthy men without type 2 diabetes [10]. For overweight or obese patients and patients with metabolic syndrome, oats fiber also improved glucose intolerance and insulin sensitivity [11,12]. However, the European Food Safety Authority (EFSA) reported that the evidence remained insufficient to prove the relationship between β-glucan consumption and the long-term maintenance of normal blood glucose level [13]. Accordingly, the aim of this systematic review was to comprehensively evaluate if oats intake is beneficial for both the short-term glucose response and the long-term glucose control as well as other metabolic parameters such as lipid and anthropometric profiles in type 2 diabetic patients.

## 2. Methods

### 2.1. Literature Search and Study Selection

The electronic database of PubMed was searched for articles published before 23 August 2015 using the keywords “oat”, “oats”, or “oatmeal” and “diabetes”. Medical Subject Heading (MeSH) was also used during the search when applicable. The references lists of original studies and review articles investigating the relationship between oats intake and diabetes were screened to make sure all potentially relevant studies were included.

Studies were included if they met the following criteria: (1) Clinical trials or observational studies; (2) Participants with type 2 diabetes mellitus; (3) Oats or oatmeal or oats-containing products as the intervention or exposure; (4) Reporting the changes of blood glucose, insulin, HbA1c, postprandial glucose and insulin responses, insulin sensitivity or β-cell function. Changes of lipid profiles, weight and BMI were additional outcomes.

### 2.2. Data Extraction

All search studies were independently reviewed by two reviewers (Q. T. and Y. L.) and disagreements were resolved through discussion with a third reviewer (S. L.). The following information was extracted from each study using a predefined form: first author, year of publication, country, participant counts, sex, age, subject type, study design, follow-up duration, baseline HbA1c and diets. The outcomes of interest include glucose and insulin profiles, HbA1c, postprandial insulin and glucose responses, β-cell function, lipid profiles, weight and BMI.

### 2.3. Quality Assessment

The modified Jadad scale was used for reporting the quality of randomized controlled trials [14]. The scores range from 0 (very poor) to 7 (very good). The seven-point quality scale includes items for randomization (described as randomized, 1 point; described randomization method, 2 points), randomization concealment (described as randomization concealment, 1 point; described concealment method, 2 points), blinding (described as blind, 1 point; described blinding method, 2 points), and follow-up (described the withdrawal in each group, 1 point). Newcastle-Ottawa Scale (NOS) was used to score the quality of observational studies [15]. The nine-point NOS assigns points for selection (4 points), comparability (2 points) and outcome (3 points).

### 2.4. Statistical Methods and Evidence Assessment

We chose a literal description and a meta-analysis to report the results. The change form baseline in each diet pattern or the change of the intervention diet relative to the control diet was displayed in the tables. Statistically significant changes (*p* < 0.05) were marked with different symbols in the tables. The meta-analysis was carried out using STATA 12.0, and the changes from baseline of metabolic parameters were calculated as the mean differences (MD) with their 95% confidence intervals (CIs). The Grading of Recommendations Assessment, Development, and Evaluation (GRADE) system (GRADEprofiler 3.6.1) was used to rate the quality of evidence.

## 3. Results

### 3.1. Search Results

**Figure 1:**
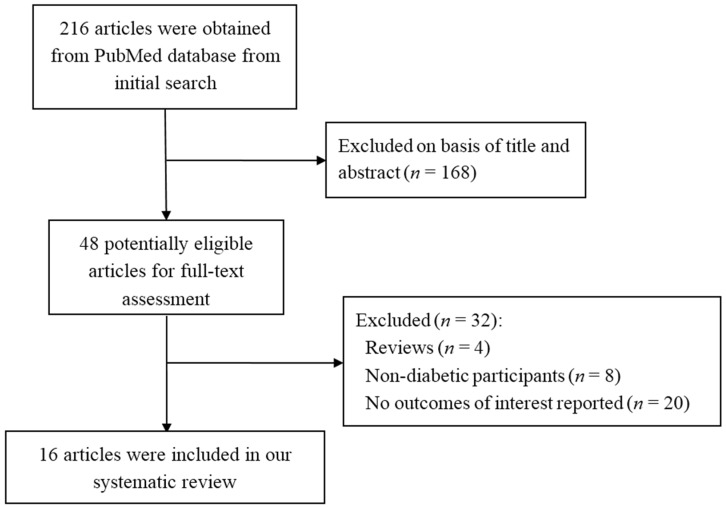
Flow diagram for study identification.

A total of 216 articles were identified (Figure 1). One hundred and sixty-eight articles were excluded after screening the titles and abstracts and forty-eight potentially eligible articles were left for full-text assessing. A further thirty-two articles were excluded for the following reasons: (1) Review articles (*n* = 4); (2) Participants were not diabetic patients (*n* = 8); (3) No outcomes of interest were reported (*n* = 20). Finally, sixteen articles [9,16,17,18,19,20,21,22,23,24,25,26,27,28,29,30] were included in this systematic review.

Fourteen controlled trials (4 paralleled designs and 10 crossover designs) [9,16,17,18,19,20,21,22,23,24,25,26,27,28] and two uncontrolled observational studies [29,30] were finally analyzed. The characteristics of the studies included in this systematic review are shown in Table 1. The detailed diet information is displayed in Table S1. Eight studies [17,18,19,22,26,27,29,30] were carried out in Europe, three studies [20,24,25] were carried out in Canada, two in China [16,23] and one in Venezuela [9], USA [21] and Mexico [28]. All the studies focused on type 2 diabetic patients, and three [9,25,27] of them only studied males. The number of subjects ranged from 8 to 260, and the follow-up duration ranged from a single-meal to twelve weeks. When we evaluated the study quality, seven studies [16,17,18,21,23,27,28] were classified as high-quality studies (modified Jadad score ≥4) and the remaining seven [9,19,20,22,24,25,26] as low-quality studies (modified Jadad score <4) (Table S2). Additionally, the two observational studies received a NOS score of 7 [29] and 6 [30], respectively (Table S3).

**Table 1:** Baseline characteristics of studies included.

Study	Country	No. of Subjects	Sex (F %)	Age (Year)	Subject Type	Design	Follow-up Duration	Baseline HbA1c (%)
Reyna, 2003 [9]	Venezuela	16	Male	45–55	Well controlled T2DM	Parallel RCT	4 weeks	8.3
Ma, 2013 [16]	China	260	M & F (56.9)	50–65	T2DM, MetS	Parallel RCT	30 days	9.9
Liatis, 2009 [17]	Greece	46	M & F (43.9)	63	T2DM	Parallel RCT	3 weeks	7.1
Cugnet-Anceau, 2009 [18]	France & Sweden	53	M & F (39.6)	30–75	Free-living T2DM	Parallel RCT	8 weeks	7.4
Tappy, 1996 [19]	Switzerland	8	M & F (12.5)	34–65	T2DM	Crossover RCT	Single meal	6.4
Jenkins, 2002 [20]	Canada	16	M & F (37.5)	46–70 (61 ± 2)	T2DM	Crossover RCT	Single meal	7.4
Rendell, 2005 [21]	USA	18	M & F (33.3)	62 ± 3	T2DM only under diet management	Crossover RCT	Single meal	NA
Tapola, 2005 [22]	Finland	12	M & F (58.3)	18–75 (66 ± 7)	T2DMonly under diet management	Crossover RCT	Single meal	NA
Yu, 2014 [23]	China	30	M & F (56.7)	48–73 (66 ± 6)	T2DM without insulin therapy	Crossover RCT	Single meal	6.8
Braaten, 1994 [24]	Canada	8	M & F (62.5)	59 (50–68)	T2DM	Non-randomised crossover trial	Single meal	8.3
Pick, 1996 [25]	Canada	8	Male	39–57 (46 ± 1)	T2DM	Crossover RCT	2 consecutive 12-week	7.0
McGeoch, 2013 [26]	UK	27	M & F (33.3)	46–71	T2DM under diet and lifestyle management	Crossover RCT	2 consecutive 8-week	6.8
Kabir, 2002 [27]	France	13	Male	41–67 (59 ± 2)	T2DM	Crossover RCT	2 periods of 4 weeks with a 15-day washout interval	8.3
Ballesteros, 2015 [28]	Mexico	29	M & F (34.5)	54 ± 8	Well controlled T2DM	Crossover RCT	2 periods of 5 weeks with a 3-week washout interval	6.8
Lammert, 2007 [29]	Germany	14	M & F (71.1)	60 ± 10	Uncontrolled T2DM, insulin resistance, MetS	Uncontrolled prospective observational study	2 days & 4 weeks	8.6
Zerm, 2013 [30]	Germany	50	M & F (52.0)	65 ± 10	Poorly controlled T2DM, insulin resistance, obese	Uncontrolled retrospective observational study	2 days	9.6

HbA1c, glycosylated hemoglobin A1c; M, male; F, female; T2DM, type 2 diabetes mellitus; RCT, randomized controlled study; MetS, metabolic syndrome; NA, not available.

### 3.2. Glucose Control and Insulin Profiles

**Figure 2:**
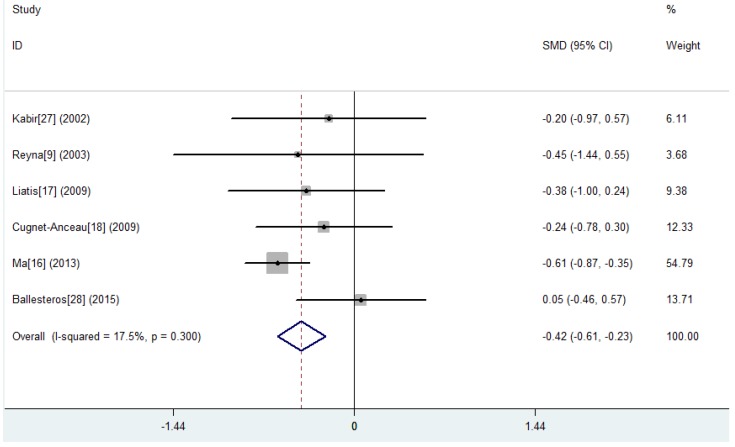
Results of the meta-analysis carried out to investigate the effect of oats intake on glycosylated hemoglobin A1c (HbA1c). The changes from baseline (Mean ± SD) between the two groups were compared. MD, mean difference; CI, confidence interval.

**Figure 3:**
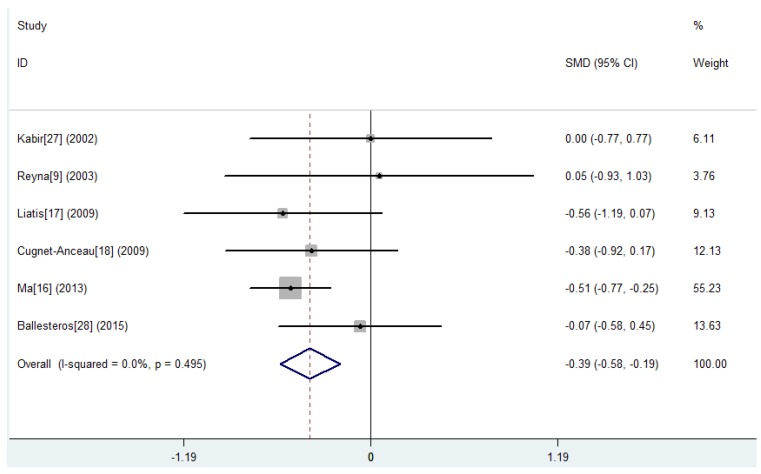
Results of the meta-analysis carried out to investigate the effect of oat intake on fasting blood glucose (FBG). The changes from baseline (Mean ± SD) between the two groups were compared. MD, mean difference; CI, confidence interval.

Table 2 shows the results of nine studies investigating the changes of glucose and insulin levels after oats interventions or exposures. Eight studies reported HbA1c. Three randomized, parallel controlled studies [9,16,17] showed a significant reduction from baseline (−0.28% to −2.22%; *p* < 0.05) in the oats intervention group and a significant reduction was observed in subjects who consumed oats than in the control subjects (MD, −0.42%; 95% CI, −0.61% to −0.23%; *p* < 0.001) (Figure 2, Table 3). Among the seven studies reporting fasting blood glucose (FBG), two [16,17] randomized, parallel controlled studies showed a significant reduction from baseline (−0.72 to −1.91 mmol/L; *p* < 0.05) in the oats intervention group. A significant reduction was observed in subjects who consumed oats than in the control subjects (MD, −0.39 mmol/L; 95% CI, −0.58 to −0.19 mmol/L; *p* < 0.001) (Figure 3, Table 3). One study showed a significantly greater reduction from baseline following oats intervention compared with the control group of usual care (*p* < 0.05) [16]. Only one randomized, parallel controlled study [16] reported the postprandial blood glucose (PBG). It showed that 50 g and 100 g of organic naked oat with whole germ (ONOG) significantly decreased the 2-h PBG by 3.25 mmol/L (*p* < 0.05) and 3.70 mmol/L (*p* < 0.05) from baseline after 30 days of an oats diet, respectively. Additionally, this reduction from baseline in the 100 g-ONOG group was statistically greater compared with the 50 g-ONOG group (*p* < 0.05). Four studies reported fasting insulin (FINS). Among them, one randomized, parallel controlled study [17] showed a non-significant reduction from baseline (−3.23 μU/mL; *p* > 0.05) after three weeks of β-glucan bread intervention and a non-significant increase from baseline (+3.77 μU/mL; *p* > 0.05) after white bread intervention. Although the changes from baseline were not significant within group, the relative changes between groups were significantly different in this study (*p* < 0.05). The pooled effect of oats intake on FINS was only from two studies (MD, −0.22 μU/mL; 95% CI, −1.28 to 0.84 μU/mL; *p* = 0.681) (Figure S1, Table 3). Two uncontrolled observational studies [29,30] investigated mean blood glucose (MBG) and mean daily insulin (MDI) changes from baseline after two days of oatmeal consumption in poorly controlled type 2 diabetic patients with insulin resistance. The MBG decreased by 1.08 to 2.39 mmol/L (*p* < 0.05), and the MDI decreased by 36.60 to 62.00 IU/day (*p* < 0.05) at different time points after the oatmeal consumption.

Four randomized studies [16,17,26,28] used the homeostasis model assessment (HOMA) of insulin resistance or β-cell function. Liatis *et al.* [17] revealed a non-significant decrease in insulin resistance from baseline (−2.08 μU × mol/L^2^; *p* > 0.05) in the β-glucan bread (3 g/day β-glucan) group and a non-significant increase from baseline (+1.33 μU × mol/L^2^; *p* > 0.05) in the white bread group. The relative changes from baseline were significantly different between the two groups (*p* < 0.05). Ma *et al.* [16] found a significant decrease in insulin resistance from baseline (−0.33 μU × mol/L^2^; *p* < 0.05) after an intervention of 100 g/day organic naked oat with whole germ (ONOG) (5.0 g/day β-glucan) based on systematic diet plans and intensive education. Whereas, the decrease in insulin resistance was not significant in the 50 g-ONOG group (−0.11 μU × mol/L^2^; *p* > 0.05). The pooled effect of oats intake on HOMA-IR was from two studies (MD, −0.51 μU × mol/L^2^; 95% CI, −1.05 to 0.02 μU × mol/L^2^; *p* = 0.061) (Figure S2, Table 3). McGeoch *et al.* [26] and Ballesteros *et al.* [28] did not find a diet-related effect on the insulin resistance or β-cell function.

**Table 2:** Glucose control and insulin profiles.

Study	Comparison	FBG (mmol/L)	PBG (mmol/L)	FINS (μU/mL)	PINS (μU/mL)	HbA1c (%)	HOMA-IR (μU × mol/L^2^)	HOMA-B (mU/mmol)
Reyna, 2003 [9]	Modified diet *V.* baseline	0.37 ↓	NA	NA	NA	0.40 ↓^§,^*	NA	NA
	ADA’s diet *V.* baseline	0.39 ↓	NA	NA	NA	0.20 ↓^§^	NA	NA
Ma, 2013 [16]	Usual care *V*. baseline	0.22 ↓	0.01 ↓	NA	NA	0.22 ↓	0.11 ↓	NA
	Diet *V*. baseline	1.18 ↓^§,a^	2.49 ↓^§,a^	NA	NA	1.71 ↓^§,a^	0.27 ↓^§^	NA
	50 g-ONOG *V*. baseline	1.64 ↓^§,a^	3.25 ↓^§,a^	NA	NA	2.21 ↓^§,a^	0.11 ↓	NA
	100 g-ONOG *V*. baseline	1.91 ↓^§,a,b^	3.70 ↓^§,a,b^	NA	NA	2.22 ↓^§,a,b^	0.33 ↓^§,a,c^	NA
Liatis, 2009 [17]	β-glucan bread *V.* baseline	0.72 ↓^§^	NA	3.23 ↓*	NA	0.28 ↓^§^	2.08 ↓*	NA
	White bread *V.* baseline	0.07 ↓	NA	3.77 ↑	NA	0.13 ↓	1.33 ↑	NA
Cugnet-Anceau, 2009 [18]	β-glucan soup *V*. baseline	0.11 ↑	NA	NA	NA	0.00 ↑	NA	NA
	Control soup *V*. baseline	0.80 ↑	NA	NA	NA	0.17 ↑	NA	NA
McGeoch, 2013 [26]	Oat-enriched diet *V*. habitual diet (baseline)	0.30 ↑	NA	0.40 ↓	NA	0.10 ↑	0.10 ↑	5.30 ↓
	Standard dietary advice *V*. habitual diet (baseline)	0.60 ↑	NA	0.00	NA	0.20 ↑	0.30 ↑	1.00 ↓
	Oat-enriched diet *V*. standard dietary advice	0.30 ↓	NA	0.40 ↓	NA	0.10 ↓	0.20 ↓	4.30 ↓
Kabir, 2002 [27]	Low-GIB (GI: 40%) *V*. baseline	0.30 ↓	NA	2.78 ↑	NA	0.50 ↓	NA	NA
	High-GIB (GI: 64%) *V*. baseline	0.30 ↓	NA	5.00 ↑	NA	0.20 ↓	NA	NA
Ballesteros, 2015 [28]	Oatmeal breakfast *V*. egg breakfast	0.20 ↓	NA	2.03 ↓	NA	0.05 ↑	0.60 ↓	NA
Lammert, 2007 [29]	After 2 days of oatmeal *V*. baseline	MBG: 2.39 ↓^§^		MDI: 62.00 U/d ↓^§^		NA	NA	NA
	4 weeks after 2 days of oatmeal *V*. baseline	MBG: 0.94 ↓		MDI: 46.80 IU/d ↓^§^		0.40 ↓	NA	NA
Zerm, 2013 [30]	Day 2 after 2 days of oatmeal *V.* baseline	MBG: 1.08 ↓^§^		MDI: 62.00 U/d ↓^§^		NA	NA	NA
	Day 3 after 2 days of oatmeal *V.* baseline	MBG: 1.42 ↓^§^		MDI: 36.60 IU/d ↓^§^		NA	NA	NA

The changes from baseline in each diet pattern or the changes of the intervention diet relative to the control diet are estimated. FBG, fasting blood glucose; PBG, postprandial blood glucose; FINS, fasting insulin; PINS, postprandial insulin; HbA1c, glycosylated hemoglobin; HOMA-IR, homeostasis model assessment of insulin resistance; HOMA-B, homeostasis model assessment of β-cell function; ADA, American Diabetes Association; NA, not available; ONOG, organic naked oat with whole germ; GIB, glycemic index breakfast; GI, glycemic index; MBG, mean blood glucose; MDI, mean daily insulin. ^§^, changes were statistically significant from baseline (*p* < 0.05); *, changes from baseline were significantly different between groups (*p* < 0.05); ^a^ *p* < 0.05, *vs.* usual care group; ^b^ *p* < 0.05, *vs.* diet group; ^c^ *p* < 0.05, *vs.* 50 g-ONOG plus diet group.

**Table 3:** Pooled effects of oats intake on metabolic parameters of type 2 diabetic patients.

		No. of Subjects				Test of Heterogeneity		
Variables	No. of Studies	Intervention Group	Control Group	MD	95% CI	*p_h_*	*I^2^* (%)	*p_z_*
FBG (mmol/L)	6	229	208	−0.39	−0.58, −0.19	0.495	0.0 *	<0.001
FINS (μU/mL)	2	36	31	−0.22	−1.28, 0.84	0.035	77.5 ^§^	0.681
HbA1c (%)	6	229	208	−0.42	−0.61, −0.23	0.300	17.5 *	<0.001
HOMA-IR (μU × mol/L^2^)	2	150	134	−0.51	−1.05, 0.02	0.107	61.6 ^§^	0.061
TC (mmol/L)	7	237	216	−0.49	−0.86, −0.12	0.016	61.7 ^§^	0.010
LDL-C (mmol/L)	5	216	195	−0.29	−0.48, −0.09	0.284	20.5 *	0.004
HDL-C (mmol/L)	6	229	208	−0.05	−0.24, 0.14	0.608	0.0 *	0.599
TG (mmol/L)	7	237	216	−0.16	−0.34, 0.03	0.351	10.2 *	0.097
Weight (kg)	3	158	142	−0.10	−0.33, 0.12	0.505	0.0 *	0.372
BMI (kg/m^2^)	4	187	166	−0.14	−0.35, 0.07	0.566	0.0 *	0.205

FBG, fasting blood glucose; FINS, fasting insulin; HbA1c, glycosylated hemoglobin; HOMA-IR, homeostasis model assessment of insulin resistance; TC, total cholesterol; LDL-C, low-density lipoprotein cholesterol; HDL-C, high-density lipoprotein cholesterol; TG, triglyceride; BMI, body mass index; MD, mean difference; CI, confidence interval. *p_h_* and *I^2^* were used for heterogeneity assessment by Cochran’s Q test, and *p_h_* < 0·1 or *I^2^* > 50% was considered to indicate significant heterogeneity across the studies. *p_z_*, *p* value for Z test. * The fixed-effects model was applied. ^§^ The random-effects model was applied.

### 3.3. Single Meal Responses of Glucose and Insulin

Table 4 shows the glucose and insulin responses after oats intake. Six crossover studies [19,20,21,22,23,24] compared the glucose or insulin responses between the single oatmeal with different amounts of β-glucan and the control meal without β-glucan. Compared with the control meal, a single meal of oatmeal significantly reduced the acute postprandial glucose or insulin responses in all six studies. Specifically, the area under the curve (AUC) and the peak of glucose after oatmeal was 11.09% to 79.41% smaller (*p* < 0.05) and 26.38% to 81.82% lower (*p* < 0.05), respectively. The AUC of insulin was 18.89% to 67.74% smaller (*p* < 0.05) and the peak of insulin was 32.72% to 83.48% lower (*p* < 0.05). A β-glucan dosage-dependent reduction in the glucose and insulin responses was observed in one study [19].

Another three crossover trials [25,26,27] reported the glucose and insulin responses after a relatively long term of oatmeal intervention. One study [25] with a follow-up duration of two consecutive 12-week periods showed the AUCs of glucose and insulin after breakfast were significantly smaller for the oat bran concentrate bread period than the white bread period (glucose AUC: 41.98% smaller; insulin AUC: 24.52% smaller; both *p* < 0.05). The insulin peak after breakfast was 15.24% lower (*p* < 0.05) in the oat bran concentrate bread period than in the white bread period. There were no statistically significant differences in the glucose and insulin responses after lunch between the two diet periods. One study [26] enrolled 27 type 2 diabetic patients only with diet and lifestyle managements, and it did not find different diet-related effects on the postprandial glucose and insulin responses between the oat-enriched diet period and the standard dietary advice period. Kabir *et al.* [27] found that the low-glycemic index breakfast (low-GIB) with 3 g of β-glucan from oats could induce lower acute postprandial glucose and insulin responses compared with the high-glycemic index breakfast (high-GIB) without β-glucan at both the beginning and the end of a four-week intervention (*p* < 0.05). However, there were no significantly chronic changes from baseline within each group (*p* > 0.05).

Data from these nine studies illustrated that a single-oatmeal can significantly reduce the acute postprandial glucose or insulin responses when compared with the control meal. However, the changes of postprandial glucose or insulin responses after a relatively long period of oat intervention were heterogeneous when compared with the same period of control food.

**Table 4:** Single meal responses of glucose and insulin.

Study	Comparison	Glucose Response		Insulin Response	
		AUC	Peak	AUC	Peak
Tappy, 1996 [19]	4.0 g *V.* 0 g β-glucan	4 h: 29.00% ↓	33.00% ↓^#^	NA	4 h:33.00% ↓^#^
	6.0 g *V.* 0 g β-glucan	59.00% ↓^#^	58.00% ↓^#^	NA	38.00% ↓^#^
	8.4 g *V.* 0 g β-glucan	65.00% ↓^#^	62.00% ↓^#^	NA	41.00% ↓^#^
Jenkins, 2002 [20]	Oat bran cereal (3.7 g β-glucan) *V*. white bread	3 h: 11.09% ↓^#^	NA	NA	NA
****	β-glucan bar (6.2 g β-glucan) *V*. white bread	55.77% ↓^#^	NA	NA	NA
****	β-glucan cereal (7.3 g β-glucan) *V*. white bread	46.78% ↓^#^	NA	NA	NA
Rendell, 2005 [21]	Prowash (9.9 g β-glucan) *V.* liquid meal replacer	3 h: 42.36% ↓^#^	59.37% ↓^#^	3 h: 67.74% ↓^#^	83.48% ↓^#^
	Prowash *V.* oatmeal (3.1 g β-glucan)	58.50% ↓^#^	64.85% ↓^#^	67.74% ↓^#^	72.83% ↓^#^
Tapola, 2005 [22]	Oat bran flour *V*. 12.5 g glucose load	1 h: 79.41% ↓^#^; 2 h: 60.17% ↓^#^	81.82% ↓^#^	NA	NA
	Oat bran crisp *V*. 12.5 g glucose load	1 h: 49.02% ↓^#^; 2 h: 21.19% ↓	45.45% ↓^#^	NA	NA
	25 g glucose load + 30 g oat bran flour *V*. 25 g glucose load	1 h: 35.00% ↓^#^; 2 h: 22.00% ↓^#^	34.00% ↓^#^	NA	NA
Yu, 2014 [23]	SDF liquid (7.5 g β-glucan) *V*. SDF-free liquid	NA	26.38% ↓^#^	NA	32.72% ↓^#^
Braaten, 1994 [24]	Wheat farina with oat gum (8.8 g β-glucan) *V*. wheat farina	3 h: 20.35% ↓^#^	26.76% ↓^#^	3 h: 18.89% ↓^#^	NA
	Oat bran (8.8 g β-glucan) *V*. wheat farina	19.95% ↓^#^	26.76% ↓^#^	8.39% ↑^#^	NA
Pick, 1996 [25]	Oat bran concentrate bread *V.* white bread	Total 8 h: 46.06% ↓^#^; breakfast (4 h): 41.98% ↓^#^; lunch (4 h): 52.07% ↓	breakfast (4 h): 12.99% ↓; lunch (4 h): 15.27% ↓	Total 8 h: 18.66% ↓; breakfast (4 h): 24.52% ↓^#^; lunch (4 h): 13.61% ↓	breakfast (4 h): 15.24% ↓^#^; lunch (4 h): 10.99% ↓
McGeoch, 2013 [26]	Oat-enriched diet *V*. habitual diet (baseline)	3 h: 8.75% ↑^§^	NA	3 h: 3.84% ↑	NA
	Standard dietary advice *V*. habitual diet (baseline)	10.92% ↑^§^	NA	3.99% ↑	NA
	Oat-enriched diet *V*. standard dietary advice	1.96% ↓	NA	0.15% ↑	NA
Kabir, 2002 [27]	Low-GIB (GI: 40%) *V*. baseline	3 h: 14.58% ↑	6.90% ↑	3 h: 10.77% ↓	8.00% ↑
	High-GIB (GI: 64%) *V*. baseline	3.66% ↑	2.00% ↑	0.00%	4.76% ↓

The percentage changes from baseline in each diet pattern or the percentage changes of the intervention diet relative to the control diet are estimated. AUC, area under the curve; NA, not available; SDF, soluble dietary fiber; GIB, glycemic index breakfast; GI, glycemic index. ^§^, changes were statistically significant from baseline (*p* < 0.05); # changes were significantly different between groups.

### 3.4. Lipid Profiles

Nine studies assessed the changes of lipid profiles after oats interventions (Table 5). Five studies [9,16,17,26,29] revealed a significant reduction in total cholesterol (TC) from baseline after oats interventions, and this reduction ranged from −0.10 to −0.80 mmol/L (−2.00 to −12.80 percent) (*p* < 0.05). Moreover, the relative reduction in TC from baseline was significantly greater in the oats intervention group than that in the control group in two randomized, parallel controlled studies (*p* < 0.05) [16,17]. One crossover study [27] showed a significantly different change in TC between compared periods even though the relative change from baseline within each period was not significant (low-GIB: −0.30 mmol/L; high-GIB: +0.20 mmol/L; both *p* > 0.05). The other two crossover studies [25,26] showed that the TC level was significantly lower in the oats intervention period than in the control food period (−0.74 and −0.20 mmol/L, respectively) (both *p* < 0.05). Overall, a significant reduction in TC was observed in subjects who consumed oats than in the control subjects (MD, −0.49 mmol/L; 95% CI, −0.86 to −0.12 mmol/L; *p* = 0.010) (Figure S3, Table 3). Eight studies reported the changes of low-density lipoprotein cholesterol (LDL-C), among which three randomized, parallel controlled studies [9,16,17] indicated a significant reduction from baseline (−0.22 to −0.66 mmol/L) (−7.30 to −15.79 percent) (*p* < 0.05). One crossover study [25] showed that the concentration of LDL-C was 0.77 mmol/L lower (*p* < 0.05) in the oat bran concentrate period than that in the white bread period. On the whole, oats intake significantly decreased LDL-C values (MD, −0.29 mmol/L; 95% CI, −0.48 to −0.09 mmol/L; *p* = 0.004) (Figure S4, Table 3). Among the nine studies reporting HDL-C, two randomized, parallel controlled studies [9,18] indicated a significant increase from baseline (+0.15 and +0.05 mmol/L, respectively) (both *p* < 0.05) in the oats intervention group. Additionally, the relative increase from baseline was significantly greater in the oats intervention group than in the control group in one study (intervention group: +0.15 mmol/L; control group: +0.01 mmol/L) (both *p* < 0.05) [9]. However, one randomized parallel controlled study [16] with two oats intervention groups showed a slight reduction in HDL-C from baseline (−0.06 and −0.08 mmol/L; both *p* < 0.05), while the HDL-C level in the usual care group was almost unaltered. Overall, oats intake did not significantly affect HDL-C concentrations (MD, −0.05 mmol/L; 95% CI, −0.24 to 0.14 mmol/L; *p* = 0.599) (Figure S5, Table 3). Nine studies reported triglyceride (TG), two randomized, parallel controlled studies [16,18] and one uncontrolled observational study [29], which showed a significant reduction from baseline (−0.12, −0.53 and −0.68 mmol/L, respectively) (all *p* < 0.05) after oats interventions. Additionally, the relative changes from baseline differed significantly between the oats intervention group and the control group in two studies (*p* < 0.05) [16,18]. On the whole, compared with the control dietary, dietary with oats did not significantly decreased the concentrations of TG (MD, −0.16 mmol/L; 95% CI, −0.34 to 0.03 mmol/L; *p* = 0.097) (Figure S6, Table 3).

**Table 5:** Blood lipids and anthropometry parameters after interventions.

Study	Comparison	TC (mmol/L)	LDL-C (mmol/L)	HDL-C (mmol/L)	TG (mmol/L)	Weight (kg)	BMI (kg/m^2^)
Reyna, 2003 [9]	Modified diet *V.* baseline	0.38 ↓^§^	0.26 ↓^§^	0.15 ↑^§,^*	0.25 ↓	3.20 ↓^§,^*	1.20 ↓^§,^*
	ADA’s diet *V.* baseline	0.17 ↓	0.03 ↓	0.01 ↑	0.34 ↓	1.50 ↓^§^	0.40 ↓^§^
Ma, 2013 [16]	Usual care *V*. baseline	0.01 ↓	0.02 ↑	0.01 ↑	0.08 ↓	0.37 ↓	0.14 ↓
	Diet *V*. baseline	0.23 ↓^§,a^	0.03 ↓	0.07 ↓^§,a^	0.41 ↓^§^	0.86 ↓^§^	0.31 ↓^§^
	50 g-ONOG *V*. baseline	0.47 ↓^§,a,b^	0.22 ↓^§,a,b^	0.06 ↓^§,a^	0.13 ↓	0.79 ↓^§^	0.28 ↓^§^
	100 g-ONOG *V*. baseline	0.59 ↓^§,a,b^	0.31 ↓^§,a,b^	0.08 ↓^§,a^	0.53 ↓^§,a,c^	1.17 ↓^§,a^	0.45 ↓^§,a^
Liatis, 2009 [17]	β-glucan bread *V.* baseline	0.80 ↓^§,^*	0.66 ↓^§,^*	0.05 ↓	0.21 ↓	1.03 ↓^§^	0.38 ↓^§^
	White bread *V.* baseline	0.12 ↓	0.11 ↓	0.03 ↓	0.06 ↓	0.39 ↓	0.12 ↓
Cugnet-Anceau, 2009 [18]	β-glucan soup *V*. baseline	0.06 ↓	0.05 ↓	0.05 ↑^§^	0.12 ↓^§,^*	NA	0.18 ↑
	Control soup *V*. baseline	0.01 ↑	0.10 ↓	0.03 ↑	0.12 ↑^§^	NA	0.36 ↑
Pick, 1996 [25]	Oat bran concentrate bread *V.* white bread	0.74 ↓^#^	0.77 ↓^#^	0.08 ↑	0.11 ↓	NA	NA
McGeoch, 2013 [26]	Oat-enriched diet *V*. habitual diet (baseline)	0.10 ↓^§^	0.10 ↓	0.00	0.16 ↑	0.30 ↑^§^	0.20 ↑^§^
	Standard dietary advice *V*. habitual diet (baseline)	0.10 ↑^§^	0.10 ↑	0.10 ↑	0.13 ↑	0.30 ↓^§^	0.10 ↓^§^
	Oat-enriched diet *V*. standard dietary advice	0.20 ↓^#^	0.20 ↓	0.10 ↓	0.03 ↑	0.60 ↑^#^	0.30 ↑^#^
Kabir, 2002 [27]	Low-GIB (GI: 40%) *V*. baseline	0.30 ↓*	NA	0.03 ↑	0.10 ↑	NA	NA
	High-GIB (GI: 64%) *V*. baseline	0.20 ↑	NA	0.03 ↓	0.20 ↓	NA	NA
Ballesteros, 2015 [28]	Oatmeal breakfast *V*. egg breakfast	0.10 ↓	0.10 ↓	0.03 ↓	0.05 ↑	0.00	0.00
Lammert, 2007 [29]	After 2 days of oatmeal *V*. baseline	0.47 ↓^§^	0.36 ↓	0.03 ↓	0.68 ↓^§^	NA	NA
	4 weeks after 2 days of oatmeal *V*. baseline	0.00	0.13 ↓	0.10 ↑	0.41 ↓	NA	NA

The changes from baseline in each diet pattern or the changes of the intervention diet relative to the control diet are estimated. TC, total cholesterol; LDL-C, low-density lipoprotein cholesterol; HDL-C, high-density lipoprotein cholesterol; TG, triglyceride; BMI, body mass index; ADA, American Diabetes Association; ONOG, organic naked oat with whole germ; NA, not available; GIB, glycemic index breakfast; GI, glycemic index. ^§^, changes were statistically significant from baseline (*p* < 0.05); *, changes from baseline were significantly different between groups (*p* < 0.05); # changes were significantly different between groups; ^a^ *p* < 0.05, *vs.* usual care group; ^b^ *p* < 0.05, *vs.* diet group; ^c^ *p* < 0.05, *vs.* 50 g-ONOG plus diet group.

### 3.5. Weight and Body Mass Index

There were six studies reporting the changes of weight or BMI. Three randomized, parallel controlled studies [9,16,17] showed a significant reduction during the follow-up of three to four weeks. The reduction range of weight and BMI was −0.32 to −0.79 kg (*p* < 0.05) and −1.20 to −0.28 kg/m^2^ (*p* < 0.05), respectively. Only one crossover study [26] found a slight increase from baseline in weight (+0.60 kg; *p* < 0.05) and BMI (+0.30 kg/m^2^; *p* < 0.05) compared with those in standard dietary advice within 8-week follow-up. The overall changes of both the weight (MD, −0.10 kg; 95% CI, −0.33 to 0.12 kg; *p* = 0.372) and BMI (MD, −0.14 kg/m^2^; 95% CI, −0.35 to 0.07 kg/m^2^; *p* = 0.205) were not significantly different between the control dietary and the dietary with oats (Figures S7 and S8, Table 3).

### 3.6. Quality of Evidence

One critical outcome and nine important outcomes were assessed by the GRADE system. The detailed information of evidence quality is presented in Table S4.

## 4. Discussion

The present systematic review of 16 studies has demonstrated a moderately beneficial effect of oats intake on glycemic control and lipid profiles in patients with type 2 diabetes. To our knowledge, this is the first systematic review of oats consumption in patients with type 2 diabetes. On the whole, this review has revealed an improvement of glucose, insulin sensitivity and lipid profiles after oats consumption. Compared with a control meal, a single meal of oatmeal also showed superiority of acute glucose and insulin responses.

Among the eight studies investigating HbA1c, three randomized, parallel controlled studies [9,16,17] showed a significant reduction in HbA1c from baseline in the oats diet group (absolute change: −0.28%, −0.40% and −2.22%, respectively). Ma *et al.* [16] revealed the greatest beneficial effect of oats intake on diabetic patients with the following features: First, compared with common oats products, naked oats maintain the most ingredients and beneficial nutrients of the whole-oat grains, which indicates naked oats might be better for patients with diabetes. Second, a relatively large sample size (260 participants) in this study seemed to be more likely to get a positive result. Third, the baseline glucose level was relatively high (mean HbA1c 9.87%, mean FBG 9.99 mmol/L, mean PBG 18.77 mmol/L). Forth, a diet with low energy, low fat and high fiber was provided to all the participants in both the intervention and the control groups, indicating oats consumption might show its benefits especially when the general energy intake was low. However, Kabir *et al.* [27] showed that adding 3 g of β-glucan from oats to a low-glycemic index breakfast with cereal, milk, bread and butter could not lead to a significant chronic changes (four week-baseline) in FBG, FINS and HbA1c. It may be due to the fact that the original study mainly aimed to evaluate the effects of a low-glycemic index breakfast on the glucose and lipid metabolism in type 2 diabetic patients. Thus, the test meal was focused on the glycemic index of food rather than the ingredients of food such as oats. Therefore, the results of this study are less meaningful for evaluating the beneficial effects of oats intake on type 2 diabetes. On the other hand, it suggests that a background diet with added oats is important for the total effect. The above evidence suggests that adding naked oats to a calorie-restricted diet might help type 2 diabetic patients to get a more obvious hypoglycemic effect especially in those with a high level of blood glucose. The amounts of β-glucan were greater than or equal to 3 g in most oats dietaries of the included studies. Tappy *et al.* [19] revealed a dosage-dependent association between the amount of β-glucan in breakfast cereal and the response of postprandial glucose. Additionally, this inverse liner relationship was more obvious at low doses of β-glucan (below 6 g). The results of this study were confirmed by previous reports, which also showed a significant dose-dependent relationship between the hypoglycemic effect and the amount or the log viscosity of oats [31,32]. These findings will help in deciding the appropriate dose of oats or β-glucan included in the whole food system. As the UK Prospective Diabetes Study (UKPDS) Group revealed, a 1% reduction in HbA1c was associated with a 21% and 14% reduction in the risk of death related to diabetes and all-cause mortality, respectively [33]. That is to say, the magnitudes of the statistically significant reduction in HbA1c in the present review would translate to a clinically significant reduction in the risk of death related to diabetes (−8.82%) and overall mortality (−5.88%).

Compared with the controls, oats intake significantly reduced the concentrations of TC and LDL-C. The findings in the present review are consistent with previous systematic reviews or meta-analyses which also showed a significant reduction in TC and LDL-C after oats or oats β-glucan consumption at the general population level [34,35,36]. This review also revealed a decreasing tendency in TG, which was omitted previously [34,36]. This decreasing tendency may partly be explained by the relatively high baseline level of TG in type 2 diabetic patients in our review. Interestingly, two oats intervention groups in one study [16] showed a slight reduction from baseline in HDL-C (−0.06 and −0.08 mmol/L, respectively; both *p* < 0.05), while two studies [9,18] showed a slight increase in HDL-C from baseline (+0.15 and +0.05 mmol/L, respectively; both *p* < 0.05). The slight reduction in HDL-C in this study may partly be due to the side effect of a low-cholesterol and saturated-fat diet as the author of the original study discovered [37]. Whether this slight reduction would produce clinical significance remains to be determined. Some inconsistent results about the effect of oats intake on HDL-C at the general population level were also reported, Tiwari *et al.* [35] revealed an increase in HDL-C after oats intake, while Thies *et al.* [34] found a non-significant effect of oats intake on HDL-C. A characteristic pattern of diabetic dyslipidemia, which consists of a mild to marked elevation of TG and low level of HDL-C [38], may partly account for the discrepancy between the general population and the diabetic patients. Therefore, further analysis is necessary to confirm the lipids (especially HDL-C and TG) changes after oats consumption in the diabetic and non-diabetic people separately. Previous evidence showed that each 1% reduction in TC or LDL-C was associated with a 2% or 1% reduction in the risk of coronary heart disease, respectively [39]. This means the effect of oats-containing diets in this review would translate to an additional 4.00 to 25.60% reduction in coronary heart disease risk due to the lipid benefits from oats intake.

Overall, oats intake was associated with a slight decrease in body weight and BMI, but the difference was not significant. To be noted, body weight increased slightly following the oat-enriched diet compared with standard dietary advice in only one study [26], with an excess total energy and the glycemic load in the oat-enriched dietary plan. It indicated that total energy as well as other dietary components should be very carefully considered during the assessment of oats consumption in patients with diabetes. 

Oats are classified as a kind of whole grain which is different from other grains. They are particularly high in soluble fiber, β-glucan and some micronutrients such as magnesium. The unique components and special physic-chemical properties largely decide the beneficial effects of oats. The beneficial effects of oats on glycemia and blood lipids are mainly related to oats β-glucan, a soluble and fermentable fiber, which cannot be decomposed and absorbed in the small intestine but can be fermented in the colon. The β-glucan is reported to increase the viscosity of food bolus, delay gastric emptying and lengthen intestinal transit time, slow the absorption of nutrients especially the carbohydrates, and enhance the satiety [6,40,41,42,43]. It was also reported that β-glucan could slow the appearance of glucose in plasma, resulting in longer-lasting insulin secretion which exert a prolonged inhibition of endogenous glucose production and lipolysis [44]. Apart from β-glucan, oats are also a rich source of magnesium, which is an important co-factor for many enzymes including enzymes involved in the metabolism of glucose and insulin. Additionally, an inverse association between magnesium in relation to type 2 diabetes was reported [45]. A group of phenolic compounds named avenanthramides have been found in oats. Avenanthramides are traditionally considered a kind of antioxidant. Some other important effects of avenanthramides, such as enhanced endothelial function and anti-inflammatory properties, were reported recently. Thus, avenanthramides as well as some other antioxidants including vitamin E from oats could synergistically contribute to the beneficial effects on diabetes and the subsequent complications such as dyslipidemia, atherosclerosis and cardio-cerebrovascular diseases [46]. The dosage, chemical structure, molecular weight (MW), solubility and viscosity are key influential factors for the health effects of oats. Additionally, the above factors are affected by the variety and growing conditions, the processing and food preparations, and even the physiological disposition of oats *in vivo* [7,47]. The mechanisms of lowing cholesterol are not very clear, but it is suggested that β-glucan can bind with bile acids and increase the intestinal viscosity, thereby decreasing cholesterol absorption and increasing fecal bile acid excretion [48]. The variety of oats may also be an important source of the heterogeneity among studies included in the present systematic review.

The argument of oats might be raised due to its potential association with asthma, coeliac disease, dermatitis and some other allergic conditions. However, another different viewpoint has indicated that the possible association may result from a wheat contamination which contains gluten. Gluten is a group of seed storage proteins of cereals. It is also widely used in food manufacturing, usually as an ingredient and processing aid, due to its viscoelastic properties [49,50,51]. Pure oats contain avenins, which are less likely to cause allergies. However, gluten is still added to most oat breads to produce the needed elasticity and structure of bread [48]. In the current review, we did not find evidence about the relationship between oats consumption and allergic reactions or diseases. Caution is still needed to add oats to the diet of wheat hypersensitive patients. It is better to use pure oats without wheat contamination. The relationship between infant exposure to oats and the development of type 1 diabetes has been thoroughly discussed recently. Introducing oats early (<4 months of age) or late (≥6 months of age) in the infancy was reported to be related to the development of type 1 diabetes [52,53]. The American Academy of Pediatrics also recommended to introduce solid foods including oats between 4 and 6 months of age [54]. For children with susceptibility to type 1 diabetes, the introduction of oats would be with great caution. Further investigation about the safety of oats consumption in diabetic patients is required.

There are several limitations in the present review. Firstly, the limited number of studies included and the small number of participants involved in each study might not have sufficient power to detect a definite effect. Secondly, we failed to find evidence of oats consumption in patients with type 1 diabetes, which has a different pathogenesis and clinical feature from type 2 diabetes. Thirdly, the safety of oats consumption was not assessed due to insufficient data.

## 5. Conclusions

In conclusion, the present systematic review has revealed a beneficial effect of oats consumption on glucose and lipid profiles in patients with type 2 diabetes, and could therefore be recommended to patients. Naked oats, having low calories, might provide more benefits and a recommendation of 3 g or more per day of β-glucan might be beneficial. The effects of oats intake on type 1 diabetes and the safety of oats consumption should also be investigated in the future.

## Supplementary Materials

Supplementary materials can be accessed at: http://www.mdpi.com/2072-6643/7/12/5536/s1. Table S1: Diets of studies included. Table S2: Methodological quality of studies included based on modified Jadad scale. Table S3: Methodological quality of studies included based on Newcastle-Ottawa Scale. Table S4. GRADE evidence profile of the metabolic effects of oats intake in patients with type 2 diabetes. Figure S1. Results of the meta-analysis carried out to investigate the effect of oat intake on fasting insulin (FINS). The changes from baseline (Mean ± SD) between the two groups were compared. MD, mean difference; CI, confidence interval. Figure S2. Results of the meta-analysis carried out to investigate the effect of oat intake on homeostasis model assessment-insulin resistance (HOMA-IR). The changes from baseline (Mean ± SD) between the two groups were compared. MD, mean difference; CI, confidence interval. Figure S3. Results of the meta-analysis carried out to investigate the effect of oat intake on total cholesterol (TC). The changes from baseline (Mean ± SD) between the two groups were compared. MD, mean difference; CI, confidence interval. Figure S4. Results of the meta-analysis carried out to investigate the effect of oat intake on low-density lipoprotein cholesterol (LDL-C). The changes from baseline (Mean ± SD) between the two groups were compared. MD, mean difference; CI, confidence interval. Figure S5. Results of the meta-analysis carried out to investigate the effect of oat intake on high-density lipoprotein cholesterol (HDL-C). The changes from baseline (Mean ± SD) between the two groups were compared. MD, mean difference; CI, confidence interval. Figure S6. Results of the meta-analysis carried out to investigate the effect of oat intake on triglyceride (TG). The changes from baseline (Mean ± SD) between the two groups were compared. MD, mean difference; CI, confidence interval. Figure S7. Results of the meta-analysis carried out to investigate the effect of oat intake on weight. The changes from baseline (Mean ± SD) between the two groups were compared. MD, mean difference; CI, confidence interval. Figure S8. Results of the meta-analysis carried out to investigate the effect of oat intake on body mass index (BMI). The changes from baseline (Mean ± SD) between the two groups were compared. MD, mean difference; CI, confidence interval.
