# Supplementary material for: The Metabolic Effects of Oats Intake in Patients with Type 2 Diabetes: A Systematic Review and Meta-Analysis
Source: Nutrients. 2015 Dec 10;7(12):10369–87. doi: 10.3390/nu7125536 (PMC4690088; doi:10.3390/nu7125536)
Supplement: Supplementary file 1 [file nutrients-07-05536-s001.docx]

Supplementary Materials: The Metabolic Effects of Oats Intake in Patients with Type 2 Diabetes: A Systematic Review and Meta-Analysis

Qingtao Hou ^1,†^, Yun Li ^2,†^, Ling Li ^3^ and Gaiping Cheng ^4^, Xin Sun ^3^, Sheyu Li ^1,^* and Haoming Tian ^1^^,^*

**Table S1.** Diets of studies included.

| **Study** | **Intervention** | **Description** | **Energy (kcal)** | **Fiber (g)** | **β-glucan (g)** | **Background Diet** |
| --- | --- | --- | --- | --- | --- | --- |
| Reyna, 2003 [9] | ADA’s diet | ADA’s nutrition recommendations  for diabetic patients | NA | NA | NA | NA |
|  | Modified diet | Modified ADA’s diet with a fat replacer  (β-glucan from oats) and sweeteners | NA | NA | NA | NA |
| Ma, 2013 [16] | Usual care | Only basic healthy advice | 2453 (daily energy) | 22.0 | 0 | Usual diet |
|  | Diet | Systematic diet plans and intensive education | 2066 (daily energy) | 30.0 | 0 | Usual diet |
|  | 50 g-ONOG | Daily ONOG replacement based on diet group | 2050 (daily energy) | 33.0 | 2.5 | Usual diet |
|  | 100 g-ONOG | Daily ONOG replacement based on diet group | 2063 (daily energy) | 36.0 | 5.0 | Usual diet |
| Liatis, 2009 [17] | White bread | Identical-looking loaves of bread without  β-glucan, made up of the same ingredients  except that the flour was wheat flour | 294 | 3.1 | 0 | Usual diet |
|  | Oat β-glucan -enriched bread | β-glucan-enriched loaves of bread, made up of wheat flour, oat flour, leavening, malt, yeast, salt and water | 204 | 21.1 | 3.0 | Usual diet |
| [Cugnet-Anceau,](http://www.ncbi.nlm.nih.gov/pubmed/?term=Cugnet-Anceau%20C%5BAuthor%5D&cauthor=true&cauthor_uid=19781120) 2009 [18] | Control soup | Frozen ready-to-eat soups with three different flavours: lentil and ham; shrimps and dill; mushroom | 258 | 4.4 | 0 | Usual diet |
|  | β-glucan soup | Control soup + soluble oat concentrate of β-glucan | 276 | 8.8 | 3.5 | Usual diet |
| Tappy, 1996 [19] | Continental breakfast | Proximal composition of the test meal | 219 | 5.0 | 0 | Usual diet |
|  | Breakfast cereal | Mixture of oat flour,  oat bran concentrate and corn starch | 208 | 9.3 | 4.0 | Usual diet |
|  | Breakfast cereal | Mixture of oat flour,  oat bran concentrate and corn starch | 224 | 14.3 | 6.0 | Usual diet |
|  | Breakfast cereal | Mixture of oat flour,  oat bran concentrate and corn starch | 238 | 18.3 | 8.4 | Usual diet |
| Jenkins, 2002 [20] | White bread | NA | 257 | 2.6 | 0 | Usual diet |
|  | Commercial oat bran  breakfast cereal | Produced by the Quaker Oats Company (Peterborough, ON, Canada) | 310 | 10.3 | 3.7 | Usual diet |
|  | Prototype  β-glucan bar | Cooked-extruded oat bran concentrate,  wheat flakes and fructose | 316 | 17.7 | 6.2 | Usual diet |
|  | Prototype  β-glucan cereal | Cooked-extruded oat bran concentrate,  wheat flakes and fructose | 303 | 14.9 | 7.3 | Usual diet |
| Rendell, 2005 [21] | Liquid  meal replacer | A commercial liquid supplement made up of protein, fat, and carbohydrate and often  used as a test meal to assess glucose tolerance | NA | 0 | 0 | Usual diet |
|  | Oatmeal | Made up of fat, protein, starch, fiber,  and low molecular weight sugar | NA | 7.0 | 3.1 | Usual diet |
|  | Prowash meal | Made up of fat, protein, starch, fiber,  and low molecular weight sugar | NA | 23.0 | 9.9 | Usual diet |
| Tapola, 2005 [22] | Oat bran flour | 61.6 g of oat bran flour + artificial  sweetener + 150 g of coffee + water | NA | 19.5 | 9.4 | Usual diet |
|  | Oat bran crisp | 29.1 g of oat bran crisp + artificial  sweetener + 150 g of coffee + water | NA | 6.3 | 3.0 | Usual diet |
|  | 25 g glucose  load + 30 g  oat bran flour | 30 g of oat bran flour + glucose solution (25 g of glucose in 250 g of water)+ 150 g of coffee | NA | 9.5 | 4.6 | Usual diet |
| Yu, 2014 [23] | SDF-free liquid | SDF-free liquid (fiber 0 g, 500 mL, 500 Kcal) | 500 | 0 | 0 | Fiber-free diet  (total fiber < 5 g/day)  for 6 days |
|  | SDF liquid | SDF liquid (oat β-glucan 7.5 g, 500 mL, 500 Kcal) | 500 | 7.5 | 7.5 | Fiber rich diet  (total fiber 35 g/day)  for 6 days |
| Braaten, 1994 [24] | Wheat farina | Porridge meals containing wheat farina | 442 | 1.5 | 0 | ≥200 g/day carbohydrate for  3 days |
|  | Wheat farina with oat gum | Porridge meals containing  wheat farina plus oat gum | 447 | 11.1 | 8.8 | ≥200 g/day carbohydrate for  3 days |
|  | Oat bran | Porridge meals containing oat bran | 516 | 20.0 | 8.8 | ≥200 g/day carbohydrate for 3 days |
| Pick, 1996 [25] | White bread | NA | 2411 (daily energy) | 19.0 | NA | Usual diet |
|  | Oat bran concentrate bread | Oat bran concentrate bread, buns, muffins | 2567 (daily energy) | 34.0 | NA | Usual diet |
| McGeoch, 2013 [26] | Habitual diet | NA | 2085 (daily energy) | 25.8 | 0 | Usual diet |
|  | Standard dietary advice | Standard dietary advice for type 2 diabetic patients | 2006 (daily energy) | 25.4 | 0 | Usual diet |
|  | Oat-enriched diet | Oat-based products (cereals,  oatcakes, bread, cereal bars) | 2093 (daily energy) | 23.9 | 3.9 | Usual diet |
| [Kabir](http://www.ncbi.nlm.nih.gov/pubmed/?term=Kabir%20M%5BAuthor%5D&cauthor=true&cauthor_uid=12077724), 2002 [27] | High-GIB (GI: 64%) | Whole wheat grains cereal,  wholemeal bread, milk, butter | 249 | 8.7 | 0 | Usual diet |
|  | Low-GIB (GI: 40%) | Oat bran concentrate cereal,  pumpernickel, milk, butter | 263 | 10.0 | 3.0 | Usual diet |
| [Ballesteros](http://www.ncbi.nlm.nih.gov/pubmed/?term=Ballesteros%20MN%5BAuthor%5D&cauthor=true&cauthor_uid=25970149), 2015 [28] | Egg breakfast | One egg + vegetables + 2 slices of bread or 2 tortillas | 313 | 26.0 | NA | Usual diet |
|  | Oatmeal breakfast | 40 g of oatmeal + 2 cups (472 mL) of lactose-free milk | 335 | 27.5 | NA | Usual diet |
| Lammert, 2007 [29] | 15 carbohydrate units of oatmeal | NA | 1100 (daily energy) | 16.2 | NA | Diabetes-adapted diet |
| Zerm, 2013 [30] | 4 carbohydrate units (80 g)  of oatmeal | NA | NA | NA | NA | Hospital  standard menus |

ADA, American Diabetes Association; NA, not available; ONOG, organic naked oat with whole germ; SDF, soluble dietary fiber; GIB, glycemic index breakfast; GI, glycemic index.

**Table S2.** Methodological quality of studies included based on modified Jadad scale.

| **Study** | **Randomization** | **Randomization Methods** | **Randomization Concealment** | **Randomization Concealment Methods** | **Blinding** | **Blinding Methods** | **Follow-up Reporting** | **Total Score** |
| --- | --- | --- | --- | --- | --- | --- | --- | --- |
| Reyna, 2003 [9] | 1 | 0 | 0 | 0 | 0 | 0 | 1 | 2 |
| Ma, 2013 [16] | 1 | 1 | 1 | 1 | 1 | 1 | 1 | 7 |
| Liatis, 2009 [17] | 1 | 1 | 1 | 1 | 1 | 1 | 1 | 7 |
| [Cugnet-Anceau,](http://www.ncbi.nlm.nih.gov/pubmed/?term=Cugnet-Anceau%20C%5BAuthor%5D&cauthor=true&cauthor_uid=19781120) 2009 [18] | 1 | 0 | 0 | 0 | 1 | 1 | 1 | 4 |
| Tappy, 1996 [19] | 1 | 1 | 0 | 0 | 0 | 0 | 1 | 3 |
| Jenkins, 2002 [20] | 1 | 1 | 0 | 0 | 0 | 0 | 1 | 3 |
| Rendell, 2005 [21] | 1 | 1 | 0 | 0 | 1 | 1 | 1 | 5 |
| Tapola, 2005 [22] | 1 | 1 | 0 | 0 | 0 | 0 | 1 | 3 |
| Yu, 2014 [23] | 1 | 1 | 1 | 1 | 1 | 1 | 1 | 7 |
| Braaten, 1994 [24] | 0 | 0 | 0 | 0 | 0 | 0 | 1 | 1 |
| Pick, 1996 [25] | 1 | 0 | 0 | 0 | 0 | 0 | 1 | 2 |
| McGeoch, 2013 [26] | 1 | 0 | 0 | 0 | 0 | 0 | 1 | 2 |
| [Kabir](http://www.ncbi.nlm.nih.gov/pubmed/?term=Kabir%20M%5BAuthor%5D&cauthor=true&cauthor_uid=12077724), 2002 [27] | 1 | 0 | 1 | 1 | 1 | 0 | 1 | 5 |
| [Ballesteros](http://www.ncbi.nlm.nih.gov/pubmed/?term=Ballesteros%20MN%5BAuthor%5D&cauthor=true&cauthor_uid=25970149), 2015 [28] | 1 | 1 | 1 | 1 | 1 | 1 | 1 | 7 |

**Table S3.** Methodological quality of studies included based on Newcastle-Ottawa Scale.

| **Study** | **Selection** | **Comparability** | **Outcome** | **Total Score** |
| --- | --- | --- | --- | --- |
| Lammert, 2007 [29] | 2 | 2 | 3 | 7 |
| Zerm, 2013 [30] | 1 | 2 | 3 | 6 |

**Table S4.** GRADE evidence profile of the metabolic effects of oats intake in patients with type 2 diabetes.

| **Quality Assessment** | | | | | | | **No of Patients** | | **Effect** | | **Quality** | **Importance** |
| --- | --- | --- | --- | --- | --- | --- | --- | --- | --- | --- | --- | --- |
|  |  |  |  |  |  |  |  |  |  |  |  |  |
| **No of Studies** | **Design** | **Risk of Bias** | **Inconsistency** | **Indirectness** | **Imprecision** | **Publication Bias** | **Dietary with Oats** | **Control** | **Relative  (95% CI)** | **Absolute** |  |  |
| **HbA1c (%) (follow-up 3–8 weeks)** | | | | | | | | | | | | |
| 6 | randomised trials | serious ^1^ | no serious inconsistency | no serious indirectness | no serious imprecision | undetected | 229 | 208 | - | MD 0.42 lower (0.61 to  0.23 lower) | ⊕⊕⊕O  MODERATE | CRITICAL |
| **FBG (mmol/L) (follow-up 3–8 weeks)** | | | | | | | | | | | | |
| 6 | randomised trials | serious ^1^ | no serious inconsistency | no serious indirectness | no serious imprecision | undetected | 229 | 208 | - | MD 0.39 lower (0.58 to  0.19 lower) | ⊕⊕⊕⊕O  MODERATE | IMPORTANT |
| **FINS (μU/mL) (follow-up 3–4 weeks)** | | | | | | | | | | | | |
| 2 | randomised trials | serious ^1^ | serious ^2^ | no serious indirectness | serious ^3^ | undetected | 36 | 31 | - | MD 0.22 lower (1.28 lower to  0.84 higher) | ⊕OOO  VERY LOW | IMPORTANT |
| **HOMA-IR (μU × mol/L2) (follow-up 3–4 weeks)** | | | | | | | | | | | | |
| 2 | randomised trials | serious ^1^ | serious ^2^ | no serious indirectness | no serious imprecision | undetected | 150 | 134 | - | MD 0.51 lower (1.05 lower to  0.02 higher) | ⊕⊕OO  LOW | IMPORTANT |
| **TC (mmol/L) (follow-up 3–12 weeks)** | | | | | | | | | | | | |
| 7 | randomised trials | serious ^1^ | serious ^2^ | no serious indirectness | no serious imprecision | undetected | 237 | 216 | - | MD 0.49 lower (0.86 to  0.12 lower) | ⊕⊕OO  LOW | IMPORTANT |
| **LDL-C (mmol/L) (follow-up 3–8 weeks)** | | | | | | | | | | | | |
| 5 | randomised trials | serious ^1^ | no serious inconsistency | no serious indirectness | no serious imprecision | undetected | 216 | 195 | - | MD 0.29 lower (0.48 to  0.09 lower) | ⊕⊕⊕O  MODERATE | IMPORTANT |
| **HDL-C (mmol/L) (follow-up 3–8 weeks)** | | | | | | | | | | | | |
| 6 | randomised trials | serious ^1^ | no serious inconsistency | no serious indirectness | no serious imprecision | undetected | 229 | 208 | - | MD 0.05 lower (0.24 lower to  0.14 higher) | ⊕⊕⊕O  MODERATE | IMPORTANT |
| **TG (mmol/L) (follow-up 3–12 weeks)** | | | | | | | | | | | | |
| 7 | randomised trials | serious ^1^ | no serious inconsistency | no serious indirectness | no serious imprecision | undetected | 237 | 216 | - | MD 0.16 lower (0.34 lower to  0.03 higher) | ⊕⊕⊕O  MODERATE | IMPORTANT |
| **Weight (kg) (follow-up 3–4 weeks)** | | | | | | | | | | | | |
| 3 | randomised trials | serious ^1^ | no serious inconsistency | no serious indirectness | no serious imprecision | undetected | 158 | 142 | - | MD 0.10 lower (0.33 lower to  0.12 higher) | ⊕⊕⊕O  MODERATE | IMPORTANT |
| **BMI (kg/m^2^) (follow-up 3–8 weeks)** | | | | | | | | | | | | |
| 4 | randomised trials | serious ^1^ | no serious inconsistency | no serious indirectness | no serious imprecision | undetected | 187 | 166 | - | MD 0.14 lower (0.35 lower to  0.07 higher) | ⊕⊕⊕O  MODERATE | IMPORTANT |

^1^ Lack of allocation concealment or blinding; Loss to follow-up and failure to adhere to the intention-to-treat principle. ^2^ Heterogeneity test: a low *p*-value and a large *I^2^*. ^3^ Small sample size. HbA1c, glycosylated hemoglobin; FBG, fasting blood glucose; FINS, fasting insulin; HOMA-IR, homeostasis model assessment of insulin resistance; TC, total cholesterol; LDL-C, low-density lipoprotein cholesterol; HDL-C, high-density lipoprotein cholesterol; TG, [triglyceride](javascript:void(0);); BMI, body mass index; MD, mean difference; CI, confidence interval.


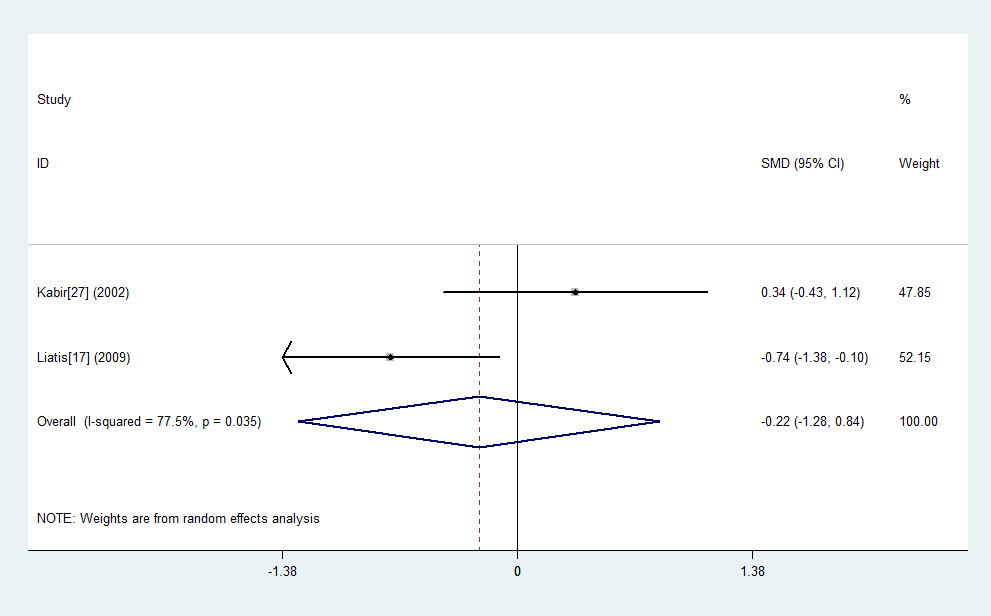


**Figure S1.** Results of the meta-analysis carried out to investigate the effect of oat intake on fasting insulin (FINS). The changes from baseline (Mean ± SD) between the two groups were compared. MD, mean difference; CI, confidence interval.


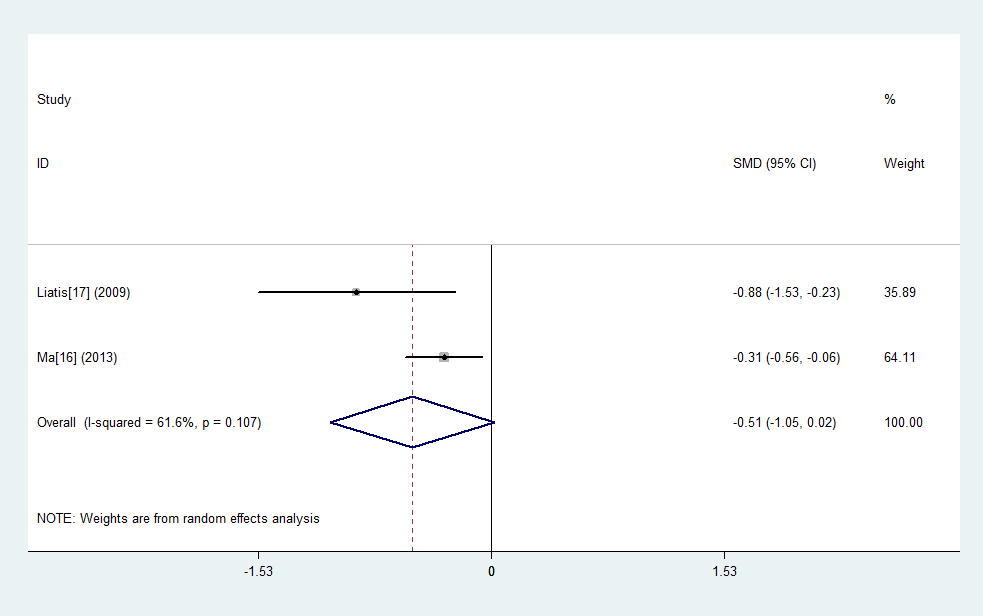


**Figure S2.** Results of the meta-analysis carried out to investigate the effect of oat intake on homeostasis model assessment-insulin resistance (HOMA-IR). The changes from baseline (Mean ± SD) between the two groups were compared. MD, mean difference; CI, confidence interval.


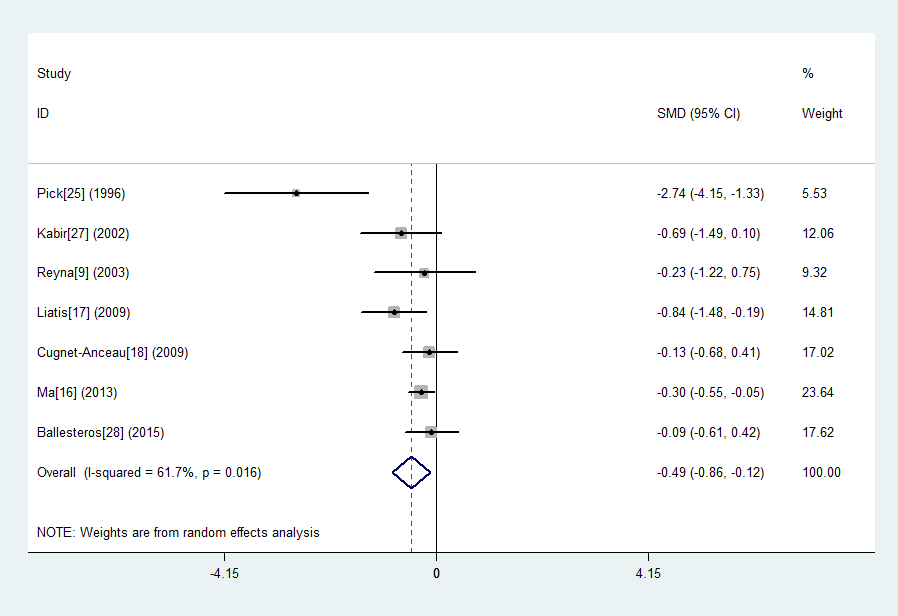


**Figure S3.** Results of the meta-analysis carried out to investigate the effect of oat intake on total cholesterol (TC). The changes from baseline (Mean ± SD) between the two groups were compared. MD, mean difference; CI, confidence interval.


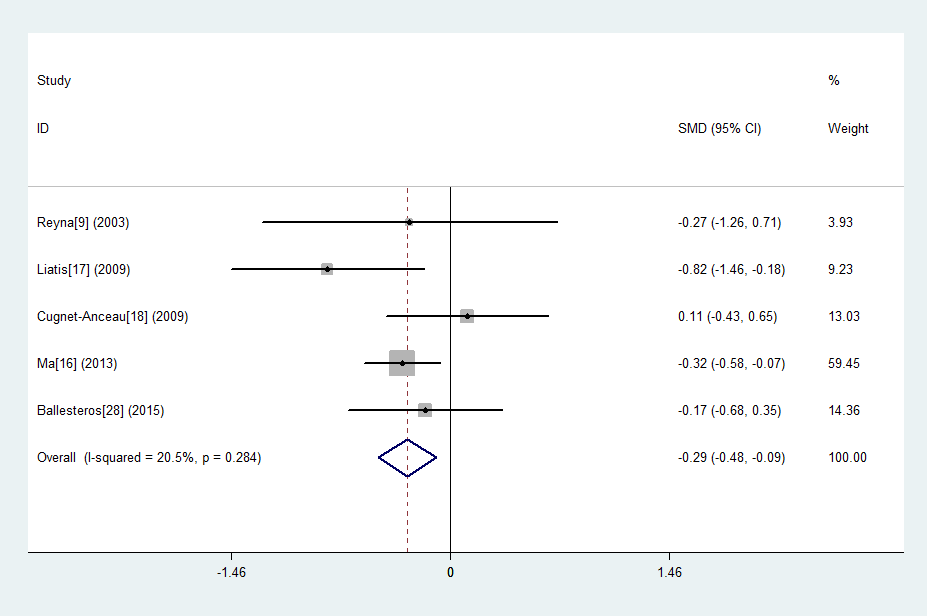


**Figure S4.** Results of the meta-analysis carried out to investigate the effect of oat intake on low-density lipoprotein cholesterol (LDL-C). The changes from baseline (Mean ± SD) between the two groups were compared. MD, mean difference; CI, confidence interval.


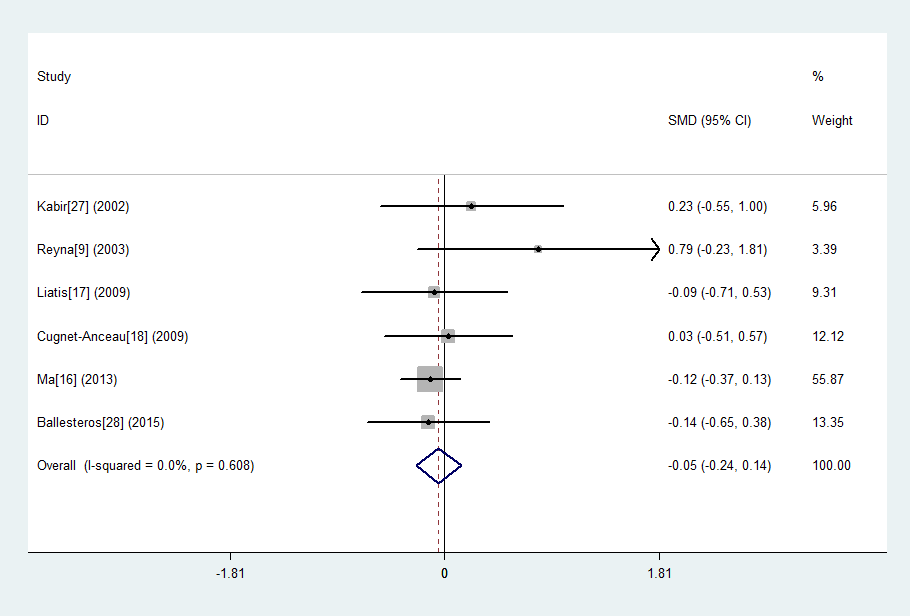


**Figure S5.** Results of the meta-analysis carried out to investigate the effect of oat intake on high-density lipoprotein cholesterol (LDL-C). The changes from baseline (Mean ± SD) between the two groups were compared. MD, mean difference; CI, confidence interval.


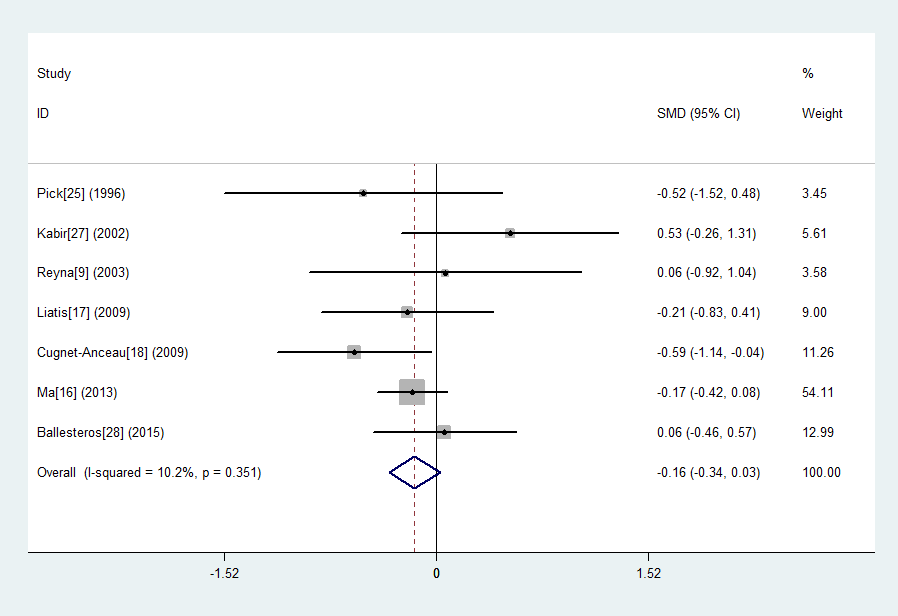


**Figure S6.** Results of the meta-analysis carried out to investigate the effect of oat intake on [triglyceride](javascript:void(0);) (TG). The changes from baseline (Mean ± SD) between the two groups were compared. MD, mean difference; CI, confidence interval.


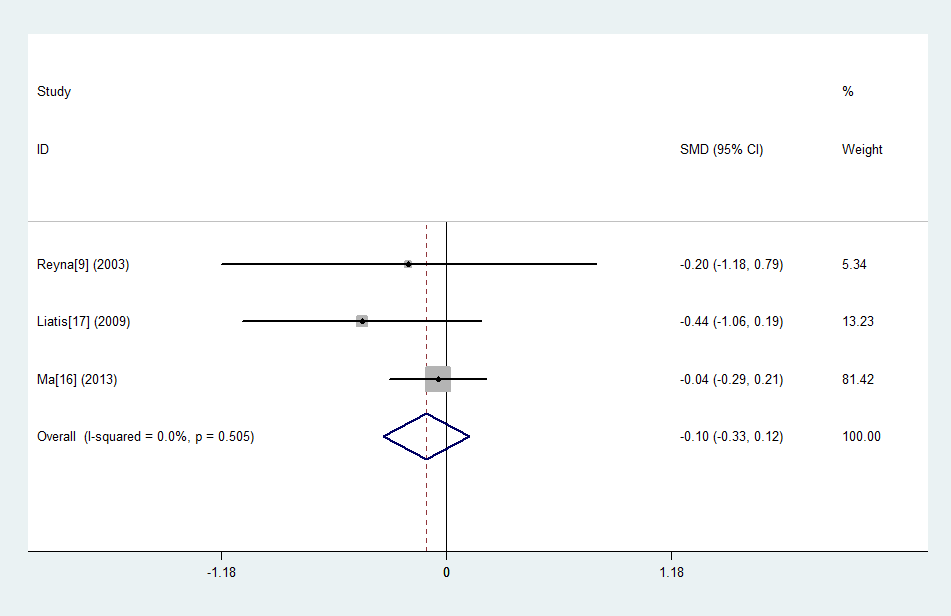


**Figure S7.** Results of the meta-analysis carried out to investigate the effect of oat intake on weight. The changes from baseline (Mean ± SD) between the two groups were compared. MD, mean difference; CI, confidence interval.


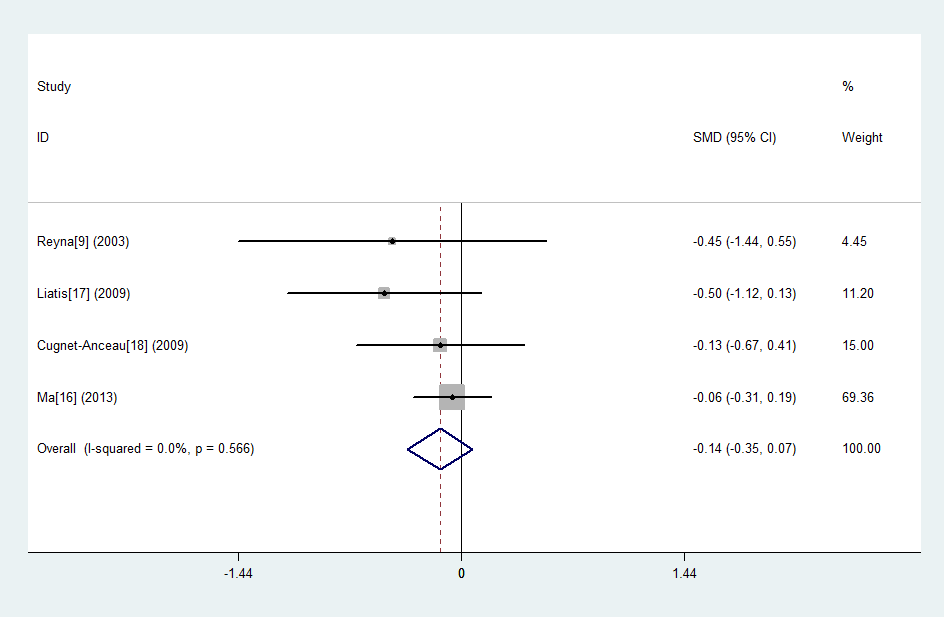


**Figure S8.** Results of the meta-analysis carried out to investigate the effect of oat intake on body mass index (BMI). The changes from baseline (Mean ± SD) between the two groups were compared. MD, mean difference; CI, confidence interval.
